# The MicroRNA Centrism in the Orchestration of Neuroinflammation in Neurodegenerative Diseases

**DOI:** 10.3390/cells8101193

**Published:** 2019-10-02

**Authors:** Nicoletta Nuzziello, Maria Liguori

**Affiliations:** National Research Council, Institute of Biomedical Technologies, Bari Unit, 70126 Bari, Italy

**Keywords:** microRNA, miRNA, neuroinflammation, competing endogenous RNAs, ceRNAs, neurodegenerative diseases, NDDs, extracellular vesicles, EVs

## Abstract

MicroRNAs (miRNAs) are small non-coding RNAs with a unique ability to regulate the transcriptomic profile by binding to complementary regulatory RNA sequences. The ability of miRNAs to enhance (proinflammatory miRNAs) or restrict (anti-inflammatory miRNAs) inflammatory signalling within the central nervous system is an area of ongoing research, particularly in the context of disorders that feature neuroinflammation, including neurodegenerative diseases (NDDs). Furthermore, the discovery of competing endogenous RNAs (ceRNAs) has led to an increase in the complexity of miRNA-mediated gene regulation, with a paradigm shift from a unidirectional to a bidirectional regulation, where miRNA acts as both a regulator and is regulated by ceRNAs. Increasing evidence has revealed that ceRNAs, including long non-coding RNAs, circular RNAs, and pseudogenes, can act as miRNA sponges to regulate neuroinflammation in NDDs within complex cross-talk regulatory machinery, which is referred to as ceRNA network (ceRNET). In this review, we discuss the role of miRNAs in neuroinflammatory regulation and the manner in which cellular and vesicular ceRNETs could influence neuroinflammatory dynamics in complex multifactorial diseases, such as NDDs.

## 1. Introduction

The eukaryotic genome is a cryptic store of genetic information [1] that encodes a large number of non-coding RNAs (ncRNAs) grouped into long ncRNAs (lncRNAs) and short ncRNAs (sncRNAs), according to their transcript size. The best studied class of sncRNAs are microRNAs (miRNAs), highly conserved and single-stranded RNAs of approximately 22 nucleotides in length, recognized as one of the key regulatory gene families in eukaryotes [2]. MiRNAs represent one of the most exciting players of modern medical sciences because they have a unique ability to modulate the transcriptomic and/or proteomic profiles.

Until recently, miRNAs were only known to display a unidirectional regulation by repressing specific protein-coding genes at the posttranscriptional level [2]. However, increasing information on the regulatory functions of miRNAs suggests that they play important roles in an extremely complex, bidirectional mechanism [3]. It has been demonstrated that the repressive actions of miRNAs are cross-regulated by competing endogenous RNAs (ceRNAs) that carry the same miRNA response elements (MREs) and can sequester miRNAs [4,5]. That concept has led to the development of complex ceRNA networks (ceRNETs), thus highlighting the complex role of miRNAs in regulating a broad range of physiological and pathological processes.

Emerging evidence supports the involvement of miRNAs as key regulators in the inflammatory response within the central nervous system (CNS), which is referred to as neuroinflammation [6]. The neuroinflammatory process is mediated by the production of proinflammatory cytokines, chemokines, secondary messengers and reactive oxygen species (ROS) [7]. Although this response can be beneficial, uncontrolled neuroinflammation can cause pathogenic tissue damage within the CNS via elevated glial cell activation, blood–brain barrier (BBB) permeability, and the infiltration of peripheral immune cells [6,8]. It is well-established that neuroinflammation plays a key pathogenetic role in several neurodegenerative diseases (NDDs), including Alzheimer’s disease (AD), Parkinson’s disease (PD), multiple sclerosis (MS), and amyotrophic lateral sclerosis (ALS) [9]. Microglial activation and the consequent release of neuroinflammatory mediators by CNS-resident cells, alongside the infiltration of immune cells into the CNS, has been detected in AD, PD, MS, and ALS [10,11,12,13]. Among the mediators of neuroinflammatory response in NDDs, miRNAs have been reported to act as enhancers or inhibitors by modulating gene expression [14,15,16,17], and related miRNA-based ceRNETs [18,19,20,21].

Additionally, miRNAs can perform their neuroinflammatory functions within the extracellular space either encapsulated within extracellular vesicles (EVs) or in an EV-free manner associated with the Ago2 protein [22], thus mediating cell-to-cell communication. Aberrantly expressed circulating miRNAs that are selectively packaged and transported in EVs to neural cells can dysregulate gene expression and/or several ceRNETs in the recipient cell, thus possibly contributing to neuroinflammatory signalling [23,24,25,26].

In this review, we draw upon existing data to highlight the role of miRNA-based neuroinflammatory regulation by focusing on emerging knowledge on ceRNETs in the modulation of neuroinflammation in NDDs. 

## 2. MiRNAs as Key Regulators of Neuroinflammation in NDDs

Neuroinflammation is defined as an inflammatory response within the CNS that aims to promote homeostasis both in physiological and pathological conditions [7]. Neuroinflammatory responses are mediated by several key proinflammatory cytokines (IL-1β, IL-6, and TNF-α), chemokines (CCL2, CCL3, CCL5, and CXCL1), secondary messengers (NO and prostaglandins), and reactive oxygen species (ROS) [27,28,29], which are produced by activated resident CNS cells, such as microglia and astrocytes [30,31,32,33]. Endothelial cells and peripherally derived immune cells are also important in propagating these inflammatory signals within the CNS [34,35]. These data suggest that neuroinflammation is a complex and well-orchestrated process involving dynamic cross-talk among heterogeneous groups of cells. A complex range of mechanisms, including negative and positive feedback loops, has evolved in nature to regulate the inflammatory process [36]. 

In this regard, the ability of miRNAs to modulate inflammatory signalling has gained considerable attention in recent years. By repressing specific targets at the posttranscriptional level, mature miRNAs can have wide-ranging effects on the function and translation of genes. The 3’ UTR mRNA targets commonly have at least one MRE region that has Watson–Crick pairing to the 5′ part of miRNA, which is located at positions 2–7 from the 5′ end of miRNA (also known as the “seed” region) [37]. Although this matching has been the basic rule for miRNA-target prediction algorithms [38], seed sites in the coding sequence (CDS) and 5’ UTR regions have also been detected, and several noncanonical MREs have been described in the literature [39,40]. Lee et al. [41] identified a new miRNA target class called miBridge, in which one miRNA simultaneously interacts with a seed pairing site in the 3’ UTR and a 3’ pairing site in the 5’ UTR. Recently, a novel class of MRE that functions exclusively in the CDS and requires extensive 3’ base-pairing and minimal 5’ base-pairing in the seed was discovered [42]. 

**Figure 1:**
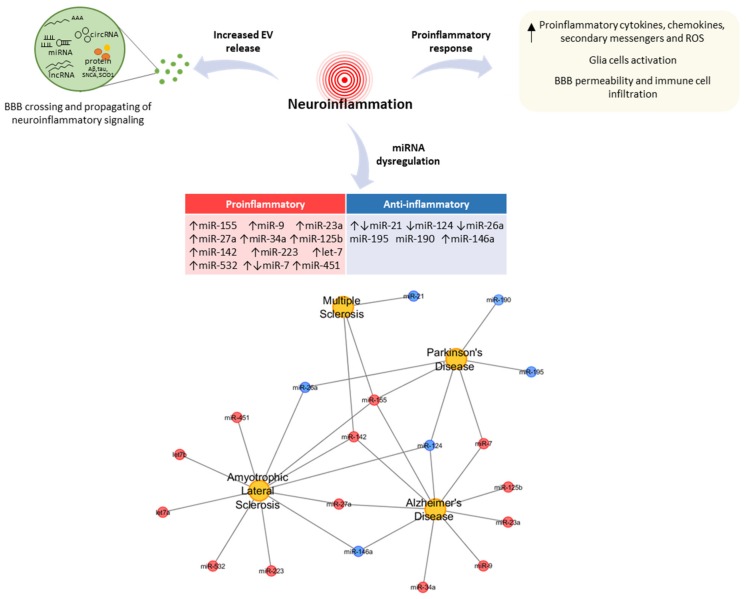
Schematic representation of the neuroinflammatory processes in neurodegenerative diseases (NDDs). CNS-resident cells suffer an activation process that leads to an increased release of extracellular vesicles (EVs) and the secretion of proinflammatory mediators, inducing the mutual activation of glial cells, BBB permeability, and the infiltration of peripheral immune cells. In the lower part, a schematic list of inflammatory miRNAs involved in NDDs is shown; the relative network was visualized using Cytoscape v3.7.1.

Depending on the mRNAs that they target, miRNAs may either promote (proinflammatory effect) or suppress (anti-inflammatory effect) neuroinflammation. Inflammatory miRNA dysregulation unavoidably affects the disorders that feature neuroinflammation, including MS, AD, PD, and ALS. Figure 1 shows some of the inflammatory miRNAs that are implicated in NDDs. 

### 2.1. Proinflammatory miRNAs: Role and Function in NDDs

MiR-155 is a key modulator of neuroinflammation and contributes to proinflammatory signalling cascades and effector functions by directly silencing the suppressor of cytokine signalling 1 (SOCS1), Fas-associated protein with death domain (FADD), IκB kinase (IKK), and interleukin 13 receptor alpha 1 (IL13Rα1), thus leading to an increase in these proinflammatory molecules [17,43]. It is considered a major inflammatory mediator that is commonly upregulated in NDDs, including MS, PD, ALS, and other motor neuron diseases, AD, and other forms of dementia. In MS, the upregulation of miR-155 has been observed in peripheral nerve and CNS-resident myeloid cells, brain lesions, blood monocytes, and stimulated microglia [15,44,45]. Consistent with a positive role for miR-155 in mediating inflammatory responses, miR-155^−/−^ mice were highly resistant to experimental autoimmune encephalomyelitis (EAE) and showed neuroprotective and attenuated neurological impairment [46]. MiR-155 is also the most upregulated miRNA in microglia and spinal cord tissue of both ALS subjects and SOD1^G93A^ mice, and its genetic ablation both restored microglia and decreased monocyte recruitment in the spinal cord of SOD1 mice [47]. Furthermore, miR-155 is the most well-studied immune-related miRNA in AD-associated neuroinflammatory events [48]. A strong upregulation of miR-155 levels was observed in the brain of a 12-month-old triple-transgenic (3xTg) AD mouse model, and this phenomenon is associated with an increase of microglia and astrocyte’s activation, and an miR-155-dependent decrease of SOCS1 [48]. It has been shown that miR-155 also regulated α-Synuclein (SNCA)-induced inflammatory responses in PD mouse models [17]. 

In a recent study, a septet of inflammatory miRNAs, including miR-155, miR-7, miR-9, miR-23a/miR-27a, miR-34a, miR-125b, and miR-146a, were found to be significantly increased in the AD neocortex [49]. The upregulation of miR-125b and miR-146a has been observed in the anatomical areas of the brain targeted by the AD process, and their role in altered neuroinflammation signalling—progressively degenerating human brain cells and tissues—is well documented [50]. Lukiw et al. [51] studied miRNA expression in the hippocampal tissue of AD patients and observed the upregulation of specific proinflammatory miRNAs, including miR-9, miR-125b, and miR-155, which all seem to be induced by NF-κB, thus indicating the possible role of these miRNAs in the neuronal inflammation of AD. 

Recent studies have reported the role of miR-142 in MS neuroinflammatory processes. Increased levels of miR-142-3p have been detected in the cerebrospinal fluid (CSF) of patients with active MS and in the brain tissue of EAE mice [52]. Furthermore, miR-142-3p was shown to regulate IL-1β-dependent synaptic abnormalities that occurred during neuroinflammation [52]. Talebi et al. [53] suggested that miR-142 isoforms could target the transcripts involved in cytokine signalling and T cell differentiation, thereby affecting the phenotype of neuroantigen-reactive T cells infiltrating the CNS during MS. The upregulation of both miR-142-3p and miR-142-5p was also reported in the prefrontal cortex of AD patients [54].

Moreover, unique proinflammatory miRNA and gene profiles were found in the blood of patients with ALS and in the microglia of mutant SOD1 mice, including increased expressions of miR-27a, miR-155, miR-146a, miR-451, miR-223, miR-142-5p, let-7a/b, and miR-532-3p, and a reduced expression of transforming growth factor beta 1 (TGF-β1) [55]. The effect of miRNAs in terms of modulating neuroinflammatory genes linked to ALS has been reported [14]. It was found that the upregulation of miR-365 and miR-125b prevented the anti-inflammatory function of the IL-3/STAT3 pathway by direct targeting. The repression of this pathway increased the activation of TNF-α gene transcription, which in turn upregulated miR-125b, thus enhancing its own transcription [14]. 

### 2.2. Anti-Anflammatory miRNAs: Role and Function in NDDs

By directly targeting mRNAs that encode specific proinflammatory mediators, miRNAs have a significant effect on the ensuing inflammatory response. The downregulated expression of anti-inflammatory miRNAs could result in the increased production of proinflammatory molecules, thus dysregulating the inflammatory response that is underlying a pathological condition. Recent studies have revealed an important role for miR-21 in the resolution of inflammation via the negative feedback of inflammatory pathways [36]. This anti-inflammatory miRNA acts as a negative modulator of toll-like receptor 4 (TLR4) signalling by targeting *programmed cell death 4* (*PDCD4*), thus leading to the reduced secretion of the proinflammatory cytokine IL-6 and the increased production of the anti-inflammatory cytokine IL-10 [56]. The role of miR-21 in MS varies by cell type and disease course, with upregulated expression in the CD4+ T cells of patients with relapsing–remitting MS [57], and downregulated expression in secondary progressive MS patients [58]. 

Several studies have indicated the anti-inflammatory role of miR-124 in neuropathologies. It has been demonstrated that miR-124 inhibited neuroinflammation in the development of PD by regulating the MEKK3/NF-κB signalling pathways [59], and by targeting p62, p38, and autophagy [60]. This anti-inflammatory miRNA was decreased in AD tissues, and consequently, its target *beta-site amyloid precursor protein cleaving enzyme 1* (*BACE1*) was increased [61]. Moreover, miR-124 was shown to promote microglial quiescence and suppress EAE by deactivating macrophages via the C/EBP-α–PU.1 pathway [62]. According to its anti-inflammatory role, miR-124 was reported to be downregulated in human leucocytes, the CSF and spinal cord tissues of ALS patients [63,64,65], and in the spinal cords and brainstems of SOD1 transgenic mice [66]. 

The role of miR-26a modulation of the inflammatory response in microglia has been investigated, wherein the overexpression of miR-26a significantly decreased the production of inflammatory cytokines, such as TNF-α and IL-6, whereas the knockdown of miR-26a increased the expression of those mediators [67]. MiR-26a-5p was downregulated in the peripheral blood of ALS patients and PD patients [68,69]. Similar to miR-26a, miR-195 overexpression inhibited the release of pro-inflammatory cytokines, including inducible nitric oxide synthase (iNOS), IL-6, and TNF-α, but induced the release of anti-inflammatory cytokines in a lipopolysaccharide (LPS)-treated microglial cell line, including IL-4 and IL-10 [70]. In the same cell line, miR-181b-5p repressed proinflammatory mediators, including TNF-α, IL-1β, and monocyte chemoattractant protein 1 (MCP1) [71]. 

Similarly, miR-190 upregulation inhibited the expression of iNOS, IL-6, and TNF-α, but increased the expression profiles of TGF-β1 and IL-10 in a PD mouse model. Moreover, miR-190 alleviated neuronal damage and inhibited neuroinflammation in a PD mouse model by targeting *nod-like receptor protein 3* (*NLRP3*), which is one of the most common inflammasomes involved in the development and progression of PD, and in the pathogenesis of various infectious and immune diseases [72]. In the midbrain of PD model mice, *NLRP3* has been reported to be a target gene of miR-7, which can inhibit microglial NLRP3 inflammasome activation when overexpressed, whereas anti-miR-7 aggravated inflammasome activation [73].

MiR-146a is another regulator of inflammation within the CNS and acts as a negative feedback regulator of NF-κB signalling by targeting the components of the *myeloid-differentiation primary response* (*MyD88*) gene’s signalling complex, including IL-1 receptor-associated kinase 1 (IRAK1), TNF receptor-associated factor 6 (TRAF6), and other signal transducers [74,75]. The inhibition of NF-κB activation leads to a decrease in the adhesion of T cells to brain endothelium, thus limiting immune cell infiltration and neuroinflammation process in NDDs [76]. Nevertheless, miR-146a has been found to be upregulated in several inflammatory NDDs [77], suggesting that this may represent a compensatory anti-inflammatory response for restoring homeostasis in the early stage of disease. 

## 3. miRNA-Based ceRNETs in the Orchestration of Neuroinflammation in NDDs

Salmena et al. [4] proposed the “ceRNA hypothesis” in 2011, stating that a bidirectional logic exists, in which transcripts can actively communicate with each other to regulate their respective expression levels via miRNAs, in addition to the conventional unidirectional miRNA-target function [78]. By acting as an RNA sponge to inhibit miRNAs from binding to their target sites, RNA transcripts that share the same MREs can competitively inhibit the function of miRNAs. On the basis of the ceRNA hypothesis, RNA transcripts communicate through a new “language” mediated by MREs to regulate their expression levels [4]. The sponge modulators include both mRNAs and ncRNAs, particularly lncRNAs, pseudogene transcripts, and circular RNAs (circRNAs) [79]. Although a large number of studies have implicated miRNAs, lncRNAs, and circRNAs as critical modulators in NDDs, the complex cross-talk involving these modulators in complex ceRNETs is still unknown and has not been studied well. 

**Figure 2:**
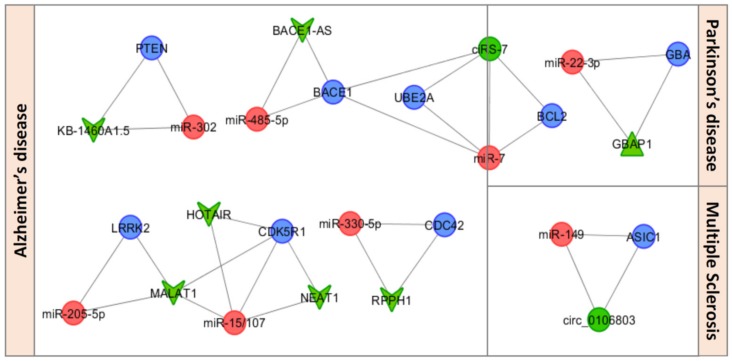
Neuroinflammatory-related ceRNETs involved in NDDs. miRNAs, competitor mRNAs and ceRNAs are represented in red, blue, and green respectively (with different shapes for each ceRNA class: circular shape for circRNAs, triangular shape for pseudogenes, and V shape for lncRNAs). The networks were visualized using Cytoscape v3.7.1.

In the following section, we will provide a brief review of ceRNETs with neuroinflammatory roles that have been investigated in NDDs (Table 1). Figure 2 shows the related ceRNA–miRNA–mRNA triple networks.

### 3.1. CircRNAs as ceRNA Regulators of Neuroinflammation in NDDs

CircRNAs are a class of endogenous ncRNAs that regulate gene expression at the transcriptional or posttranscriptional level by interacting with miRNAs, thus acting as modulators of ceRNA cross-talk [18]. Emerging evidence indicates that the circRNA–ceRNA machinery is actively involved in the pathogenesis of NDDs, but its influence on neuroinflammatory processes is still mostly unknown. 

Hansen et al. [80] identified a circRNA that was highly expressed in human and mouse brains and acted as an miR-7 sponge (referred to as ciRS-7). In the hippocampi and cortexes of AD patients, ciRS-7 downregulation was found to be coupled with an increase in miR-7, which has a known role in inflammatory degeneration, resulting in the downregulation of miR-7 target genes, such as *ubiquitin conjugating enzyme E2A* (*UBE2A*) [81]. This ciRS-7/miR-7/UBE2A circuit was found to be significantly dysregulated in the sporadic AD neocortex [81]. Notably, ciRS-7 has been shown to promote β-secretase βAPP-cleaving enzyme 1 (BACE1) degradation in an NF-κB dependent manner in AD patients [82]. BACE1 can promote inflammation via the production of two proinflammatory agents; namely, amyloid β-peptides (Aβ) and prostaglandin E2 [83]. The immune response in the brain that follows Aβ deposition results in the accumulation of inflammatory mediators, including free radicals, IL-1, IL-6, and TNF-α, and in the activation of microglia [84]. CiRS-7 was also found to be involved in PD [80,85], wherein the ciRS-7/miR-7 axis regulated B-cell lymphoma 2 (BCL2), which is known to inhibit the activation of NF-Κb and upregulate proinflammatory genes.

Several circRNA-associated ceRNETs were involved in the regulation of high mobility group protein B2 (HMGB2) by let-7g-3p in the AD mouse brain [86]. HMGB2 was reported to regulate the expression of low-density lipoprotein receptor-related protein 1 (LRP1) [87], which is known to modulate the inflammatory response [88]. Wang et al. [89] identified that 555 circRNAs, 183 miRNAs, and 319 mRNAs were significantly dysregulated in the hippocampus of AD rats, and several transcripts were thought to be associated with neuroinflammation in AD pathogenesis. 

Hsa_circ_0106803 was reported to regulate the expression of *acid-sensing ion channel 1* (*ASIC1*) by sponging miR-149 to modulate the progression of MS [90]. ASIC1 contributes to axonal degeneration in EAE mice with CNS inflammatory lesions [91]. Recently, circ-HIPK2 has been reported to increase *sigma nonopioid intracellular receptor 1* (*SIGMAR1*) expression levels by sponging endogenous miR-124-2hg, thus resulting in the significant stimulation of astrocytes. The inhibition of circ-HIPK2 can restrict activated astrocytes and could provide novel therapeutic strategies for neuroinflammation lesions [92].

### 3.2. LncRNAs as ceRNA Regulators of Neuroinflammation in NDDs

LncRNAs are a highly heterogeneous class of RNA molecules of more than 200 nucleotides in length with no protein-coding capacity. They are involved in the control of gene expression at multiple levels, such as nuclear architecture, transcription regulation, mRNA splicing, and mRNA stability. Increasing evidence has revealed that lncRNAs can act as ceRNAs via competitively sponging miRNAs to regulate neuroinflammation in several NDDs. 

Cai et al. [93] constructed the first AD-associated ceRNET by using data from the whole transcriptome and miRNA sequencing of the cortex of a transgenic mouse model, wherein they identified four hub lncRNAs, five miRNAs, and 1082 mRNA targets. The upregulated lncRNA ribonuclease P RNA component H1 (RPPH1) bound to miR-330-5p and caused the release of the downstream target *cell division control protein 42 homolog* (*CDC42*), thus leading to its upregulation and to the increase in dendritic spine density [93]. RPPH1/miR-330-5p/CDC42 ceRNET may be involved in the compensatory behaviour of brain neurons when combating synaptic loss in the early stage of AD pathogenesis. Indeed, anti-inflammatory treatments in a preclinical mouse model of AD successfully resolved the increase in spinal density, thus allowing for the establishment of novel neural connections [94]. 

An AD-associated lncRNA–miRNA–mRNA triple network was recently built. In that network, the lncRNA KB-1460A1.5 contained the MREs for miR-302, inhibited phosphatase and tensin homolog (PTEN) and activated protein kinase B (Akt) signalling, and subsequently reduced Aβ-induced neurotoxicity [95]. A significant loss and an altered distribution of PTEN, which is the major regulator of Akt, were detected in AD neurons. Notably, the Akt/PTEN pathway affected the key players of inflammation and oxidative stress involved in AD pathology [96]. 

BACE1 antisense transcript (BACE1-AS), which is an overexpressed lncRNA in several brain regions of AD patients, was found to regulate *BACE1* mRNA’s stability by masking the binding site for miR-485-5p and preventing the miRNA-induced translational repression of BACE1 [97]. Recently, nuclear enriched abundant transcript 1 (NEAT1), metastasis-associated lung adenocarcinoma transcript 1 (MALAT1), and HOX antisense intergenic RNA (HOTAIR) have been reported as lncRNAs that regulate *cyclin-dependent kinase 5 regulatory subunit 1* (*CDK5R1*) gene via miR-15/107 family in AD brain tissue [98]. NEAT1 displayed increased expression levels in the temporal cortex and hippocampus of AD patients. The overexpression of NEAT1 has been reported in other NDDs, including MS [99], PD [100], and ALS [101]. NEAT1 overexpression positively regulated the immune signalling and the expression of a group of cytokines and chemokines, including IL-6 and CXCL10, but repressed IL-1β and TNF-α production [102]. 

The MALAT1-mediated ceRNA mechanism is one of the most studied and well-characterized ceRNETs in PD. In a PD model cell line, the lncRNA MALAT1 promoted neuronal degeneration and upregulated *leucine-rich repeat kinase 2* (*LRRK2*) expression by competitively binding to miR-205-5p [103]. It has been demonstrated that LRRK2 is a positive regulator of inflammation in murine microglia, and that LRRK2 mutations may alter the microenvironment of the brain to favour neuroinflammation [104]. Furthermore, MALAT1 enhanced SNCA stability and acted as an endogenous trigger, inducing a strong inflammatory response in PD [105]. Liu et al. [106] demonstrated that MALAT1 also contributed to the apoptosis of dopaminergic neurons by sponging miR-124 within an in vitro model, and an in mouse model of PD. MALAT1 has recently been reported to promote the inflammatory response in microglia via MyD88/IRAK1/TRAF6 signalling pathway [107] and via miR-199b/IKKβ/NF-κB signalling, and to promote the production of proinflammatory cytokines (TNF-α and IL-1β) by acting as a ceRNA for miR-199b [108]. 

Two dysregulated lncRNAs in PD, namely, U1 and RP11-462G22.1, were predicted to act as ceRNAs. By using a support vector machine-learning algorithm, U1 was predicted to bind eight different miRNAs, including miR-188-3p, which controls dendritic plasticity and synaptic transmission, and miR-125b, which promotes neuronal differentiation and inflammation. RP11-462G22.1 (also known as lnc-FRG1-3) was predicted to target 21 different miRNAs, including miR-25-5p [109].

A recent study demonstrated that Sulfasalazine, an anti-inflammatory and immune-modulating drug that improves the outcome of MS patients, inhibited AKT2/NF-κB axis via the ceRNA effect of miR-136-5p and lncRNA HOTAIR in a mouse model of cuprizone-induced demyelination [110]. The AKT/NF-κB pathway has been shown to regulate the production of inflammatory cytokines in macrophages, and HOTAIR was found to be involved in the inflammatory response, which contributes to MS pathogenesis [111]. 

### 3.3. Pseudogenes as ceRNA Regulators of Neuroinflammation in NDDs

Pseudogenes are the best ceRNA candidates because they have high-sequence identity with the ancestral gene; consequently, they share common MREs with their parent genes that are competing for the same miRNAs. 

Straniero et al. [112] explored the ceRNA-based network involving the *glucocerebrosidase* gene (*GBA*) and *GBA* pseudogene (*GBAP1*), which is highly homologous (96% sequence identity) and located 16 kb downstream of the functional gene. GBAP1 was reported to function as a ceRNA that regulated GBA expression by sponging miR-22-3p in induced pluripotent stem cells (iPSCs) and iPSC-derived neurons obtained from the fibroblast sof PD patients who carried GBA mutations [112]. The mechanism by which GBA mutations increase the risk and progression of PD is still unknown; SNCA accumulation, oxidative stress, and neuroinflammation may play an important role in both the development and progression of GBA-mutant PD [113]. The dominant pathologic feature in the GBA mutant mice was a multisystem inflammatory reaction with inflammatory cell infiltration in several organs, and elevated TNF-α and IL-1β mRNA expressions [114]. 

To evaluate the potential effect of pseudogene-associated ceRNETs in NDDs, pseudogenes and their target genes that were dysregulated in NDDs were investigated, disclosing in silico predicted miRNAs binding sites that are common to pseudogene/gene pairs [115]. Although no identified ceRNET has been investigated, it is worth noting that protein phosphatase 1 regulatory inhibitor subunit 2 pseudogene 1 (PPP1R2P3) and polyhomeotic homolog 1 pseudogene 1 (PHC1P1) contained 58 and 47 MREs, respectively, and shared more than 35 MREs with their parent genes, thus causing them to behave as potent ceRNAs. These pseudogenes, as well as other ceRNA species, can potentially use the different MREs to talk with many other genes in a combinatory mechanism that is still largely unknown. 

## 4. The Neuroinflammatory Role of EVs in NDDs 

All CNS cells, including neurons, microglia, astrocytes, endothelial cells, and oligodendrocytes, release several types of EVs designated by exosomes (EXOs) and microvesicles (MVs) into the extracellular environment, depending on their biogenesis, release mechanisms, size, and surface biomarkers. EV is the collective term for cell-secreted phospholipid bilayer-bound structures that are packed with several components, including proteins, lipids, DNA, mRNAs, lncRNAs, miRNAs, circRNAs, and other sncRNAs, and serve as vehicles for the transfer and delivery of their contents between cells [116]. Although EVs regulate physiological brain functions, they are also involved in the pathogenesis of many neuroinflammatory disorders and NDDs, which trigger inflammation by carrying damage-associated molecular patterns and contribute to the propagation of neuroinflammatory signals. A significant increase in circulating EV concentration has been reported in many NDDs. Recent advances in the field of CNS-derived EVs provide the possibility of measuring the inflammatory status in the CNS without brain biopsy because EVs could directly reflect the situation in the CNS. Verderio et al. [117] reported for the first time, the release of EVs from microglia and considered their presence and concentration in the CSF of MS patients as a biomarker of inflamed CNS. 

Experimental evidence indicated that serum-derived EXOs from LPS-treated mice enhanced microglial and astrocytic activation and increased proinflammatory cytokine expression in the brain, suggesting that EXOs could mediate the activation of neuroinflammatory processes during systemic peripheral inflammation [118]. It is well known that EVs can cross the BBB and may contribute to the spread of neuroinflammatory modulators from the centre to the periphery and vice versa. In response to inflammation, EXOs induce the breakdown of the BBB, thus facilitating immune and myeloid cell transmigration and the propagation of neuroinflammatory signalling [119].

EVs have been linked to the spread of pathogenic misfolding proteins, including Aβ and tau in AD, SNCA in PD, and SOD1 in ALS, and to the triggering of an inflammatory cascade [120]. In addition to the propagation of toxic proteins, EVs also carry several inflammatory modulators, such as cytokines. It has been reported that IL-6 levels in astrocyte-derived EXOs reflected neuroinflammatory status and predicted disease progression in ALS and AD [121,122]. Increased levels of circulating EXOs are observed in EAE, where proinflammatory cytokines promoted vesicle release, which in turn spread inflammation. The injection of microglia-derived MVs into the brains of EAE mice resulted in enhanced neuroinflammation, whereas mice impaired in MV shedding were protected from EAE [117]. 

Finally, increasing evidence suggests that miRNAs that are selectively packaged into EVs and are carried to CNS-resident cells may alter the gene expression of the recipient cells and contribute to the propagation phase of neuroinflammation. The main advantage of EVs as miRNA delivery vehicles is that they are protected from the environment by their lipid bilayer, thus increasing the probability of EVs reaching their target cells.

### 4.1. EV-Associated miRNAs as Mediators of Neuroinflammation in NDDs 

The finding that miRNAs packaged into EVs can exert posttranscriptional regulatory functions in recipient cells has significantly extended their biological relevance. EVs are naturally engineered for the selective loading of miRNAs, with a programmed function for short and long-distance communication between cells. It has been shown that miR-155 and miR-146a, which are critical miRNAs that regulate neuroinflammation, were released from dendritic cells (DCs) within EXOs and mediated target gene repression in recipient DCs; miR-155 and miR-146a enhanced and reduced proinflammatory gene expression, respectively [26]. Prada et al. [23] showed that inflammatory microglia produced EVs that were enriched in a set of miRNAs that regulated the expression of key synaptic genes. Among them, miR-146a, which is a microglia-specific miRNA absent in hippocampal neurons, silenced presynaptic synaptotagmin1 (SYT1) and postsynaptic neuroligin1 (NLG1) for receiving neurons and in regard to their influence over dendritic spine formation and synaptic stability [23]. 

Extracellular miR-101 decreased in the CSF of AD patients and was correlated with increased plaque density; therefore, it contributes to neuroinflammation via the upregulation of cyclooxydenase 2 (COX2) expression, which is involved in the inflammation response in several NDDs [123]. 

Anti-inflammatory miR-124-3p is increased in microglial EXOs after traumatic brain injury; therefore, this miRNA inhibits neuronal inflammation and contributes to neurite outgrowth via the EXOs transfer into neurons [124]. Extracellular let-7, which is abundant in EXOs, has been shown to activate TLR7 and contribute to neurodegeneration [125]. Furthermore, EV-associated miR-21 was upregulated during inflammation in the brain and exhibited neurotoxicity upon the neuronal activation of the TLR7 signalling pathway [126], but it could exert anti-inflammatory effects on microglia. Notably, TLR activity has been associated with ALS, AD, and PD, and the relationship between EV-miRNAs and inflammation signalling triggered by TLRs in neighbouring cells or during long-distance cell-to-cell communication has been investigated [127]. 

Pusic et al. [128] reported that the stimulation of DCs with interferon (IFN)-γ induced the production of EXOs enriched in anti-inflammatory miRNAs compared with unstimulated DC-EXOs. Among the enriched miRNAs, miR-181a dampened proinflammatory signalling in monocyte/macrophage responses and regulated inflammation in CNS [128]. Furthermore, high levels of miR-124, miR-27a, miR-451, and miR-532-5p were found in IFNγ-DC-EXOs. Among them, miR-124 promoted anti-inflammatory signalling by downregulating IL-6, TNF-α, and iNOS. Notably, IFNγ-DC-EXOs reduced inflammation and oxidative stress and effectively increased myelin basic protein (MBP) levels in MS; this suggests a potential therapeutic role in the promotion of remyelination in MS [128]. Proinflammatory miRNAs, including miR-9, miR-125b, miR-146a, and miR-155, have been shown to be upregulated in AD CSF and extracellular fluid [77]. Those miRNAs are derived from NF-κB-regulated pre-miRNA transcripts, and their upregulation is implicated in modifying the innate immune and inflammatory response in AD brains [77]. 

### 4.2. The Emerging Role of Circulating ceRNETs 

In addition to miRNAs, EVs also contain lncRNAs and circRNAs that act like ceRNAs to regulate miRNA function in recipient cells. These ceRNAs are packaged into EVs, and their specific sorting might have two effects: (i) to discard competitors, which modifies the bioavailability of the related active miRNAs, and (ii) to transfer the competitors into recipient cells in which the active miRNA amount should be altered [129].

The role of ceRNAs carried by EVs has been mainly studied in the field of cancer research; only a few EV-ceRNAs have been reported in NDDs. A recent study revealed that circRNAs were enriched in EXOs compared with producer cells and that the ratio of circRNA level to linear RNA level in EXOs was approximately six-fold higher than that in cells [130]. Interestingly, the above mentioned ciRS-7, which is one of the most well-known circRNAs, has been reported in EXOs, and miR-7 upregulation in cells was associated with significantly reduced ciRS-7 levels in EXOs [131].

The lncRNAs RP11-462G22.1 and PCA3 were found to be upregulated in EXOs derived from the CSF of AD patients [132]. The exosomal lncRNA MALAT1 has been reported to drive regenerative function and modulate inflammation-linked networks following traumatic brain injury, by regulating the expression of mRNAs and ncRNAs involved in the inflammatory response in recipient cells [133]. 

## 5. Conclusions 

In this review, we focused on the potential effects of miRNAs on neuroinflammatory regulation in NDDs. Based on the reported literature, we hypothesized a complex cross-talk network in which miRNAs may represent the main modulators in the orchestration of neuroinflammation. The canonical unidirectional role of miRNAs can be overcome by replacing it with a more complex bidirectional mechanism, wherein miRNA acts both as a regulator and is regulated via ceRNAs in human processes. Interestingly, the function of miRNA sponges enables ceRNAs to control their activity and indirectly regulate target mRNA stability. Investigations on the actual competing capabilities of the majority of ceRNAs in neuroinflammation in NDDs are still in infancy. They may represent key players in the regulation of neuroinflammatory miRNAs because ceRNAs are evolutionarily selected to protect their cognate coding mRNAs. 

Further studies should be performed to reveal the role of miRNAs and ceRNETs in regulatory machinery. In our opinion, a future research direction in this field should also address the enzymes involved in the biogenesis of ceRNET members. Indeed, alterations in the enzymes’ structures or/and functions may possibly explain ceRNET dysregulation in NDDs that feature neuroinflammation.

## Figures and Tables

**Table 1:** Neuroinflammatory-related ceRNA networks (ceRNETs) reported in NDDs. For each ceRNET, the competing member (circRNA, lncRNA, or pseudogene), competitor, and shared miRNA are indicated.

ceRNET Type	Competing Member	Shared miRNA	Competitor (mRNA)	ceRNA Role	Ref
circRNA-miRNA-mRNA	ciRS-7	miR-7	UBE2A	Dysregulated in AD	[81]
circRNA-miRNA-mRNA	ciRS-7	miR-7	BACE1	Dysregulated in AD	[82]
circRNA-miRNA-mRNA	ciRS-7	miR-7	BCL2	Dysregulated in PD	[80,85]
circRNA-miRNA-mRNA	mm10_circ_0027470 mm10_circ_0011311 mm10_circ_0018430 mm10_circ_0009478 mm10_circ_0010326 mmu_circ_0001442	let-7g-3p	HMGB2	Dysregulated in AD	[86]
circRNA-miRNA-mRNA	hsa_circ_0106803	miR-149	ASIC1	Progression of MS	[90]
circRNA-miRNA-mRNA	hsa_circ-HIPK2	miR-124-2hg	SIGMAR1	Astrocyte activation	[92]
lncRNA-miRNA-mRNA	RPPH1	miR-330-5p	CDC42	Dysregulated in AD	[94]
lncRNA-miRNA-mRNA	KB-1460A1.5	miR-302	PTEN	Dysregulated in AD	[95]
lncRNA-miRNA-mRNA	BACE1-AS	miR-485-5p	BACE1	Dysregulated in AD	[97]
lncRNA-miRNA-mRNA	MALAT1 NEAT1 HOTAIR	miR-15/107	CDK5R1	Dysregulated in AD	[98]
lncRNA-miRNA-mRNA	MALAT1	miR-205-5p	LRRK2	Dysregulated in AD	[103]
lncRNA-miRNA-mRNA	MALAT1	miR-199b	Proinflammatory cytokines	Microglia	[108]
lncRNA-miRNA-mRNA	HOTAIR	miR-136-5p	AKT2/NF-κB axis	cuprizone-induced demyelination	[110]
pseudogene-miRNA-mRNA	GBAP1	miR-22-3p	GBA	Involved in PD	[112]
